# CXCL12-CXCR4 Interplay Facilitates Palatal Osteogenesis in Mice

**DOI:** 10.3389/fcell.2020.00771

**Published:** 2020-08-21

**Authors:** Nanne Verheijen, Christiaan M. Suttorp, René E. M. van Rheden, Raymond F. Regan, Maria P. A. C. Helmich, Anne Marie Kuijpers-Jagtman, Frank A. D. T. G. Wagener

**Affiliations:** ^1^Department of Dentistry – Orthodontics and Craniofacial Biology, Radboud University Medical Center, Nijmegen, Netherlands; ^2^Radboud Institute for Molecular Life Sciences, Radboud University Medical Center, Nijmegen, Netherlands; ^3^Department of Emergency Medicine, University of Maryland School of Medicine, Baltimore, MD, United States; ^4^Department of Orthodontics, University of Groningen, University Medical Center Groningen, Groningen, Netherlands; ^5^Department of Orthodontics and Dentofacial Orthopedics, University of Bern, Bern, Switzerland; ^6^Faculty of Dentistry, Universitas Indonesia, Jakarta, Indonesia

**Keywords:** embryology, cranial neural crest cells, Sox9, osteogenesis, palatogenesis, CXCL12-CXCR4, pathological pregnancy, heme oxygenase

## Abstract

Cranial neural crest cells (CNCCs), identified by expression of transcription factor Sox9, migrate to the first branchial arch and undergo proliferation and differentiation to form the cartilage and bone structures of the orofacial region, including the palatal bone. Sox9 promotes osteogenic differentiation and stimulates CXCL12-CXCR4 chemokine-receptor signaling, which elevates alkaline phosphatase (ALP)-activity in osteoblasts to initiate bone mineralization. Disintegration of the midline epithelial seam (MES) is crucial for palatal fusion. Since we earlier demonstrated chemokine-receptor mediated signaling by the MES, we hypothesized that chemokine CXCL12 is expressed by the disintegrating MES to promote the formation of an osteogenic center by CXCR4-positive osteoblasts. Disturbed migration of CNCCs by excess oxidative and inflammatory stress is associated with increased risk of cleft lip and palate (CLP). The cytoprotective heme oxygenase (HO) enzymes are powerful guardians harnessing injurious oxidative and inflammatory stressors and enhances osteogenic ALP-activity. By contrast, abrogation of HO-1 or HO-2 expression promotes pregnancy pathologies. We postulate that Sox9, CXCR4, and HO-1 are expressed in the ALP-activity positive osteogenic regions within the CNCCs-derived palatal mesenchyme. To investigate these hypotheses, we studied expression of Sox9, CXCL12, CXCR4, and HO-1 in relation to palatal osteogenesis between E15 and E16 using (immuno)histochemical staining of coronal palatal sections in wild-type (wt) mice. In addition, the effects of abrogated HO-2 expression in HO-2 KO mice and inhibited HO-1 and HO-2 activity by administrating HO-enzyme activity inhibitor SnMP at E11 in wt mice were investigated at E15 or E16 following palatal fusion. Overexpression of Sox9, CXCL12, CXCR4, and HO-1 was detected in the ALP-activity positive osteogenic regions within the palatal mesenchyme. Overexpression of Sox9 and CXCL12 by the disintegrating MES was detected. Neither palatal fusion nor MES disintegration seemed affected by either HO-2 abrogation or inhibition of HO-activity. Sox9 progenitors seem important to maintain the CXCR4-positive osteoblast pool to drive osteogenesis. Sox9 expression may facilitate MES disintegration and palatal fusion by promoting epithelial-to-mesenchymal transformation (EMT). CXCL12 expression by the MES and the palatal mesenchyme may promote osteogenic differentiation to create osteogenic centers. This study provides novel evidence that CXCL12-CXCR4 interplay facilitates palatal osteogenesis and palatal fusion in mice.

## Introduction

In the process of craniofacial development, cranial neural crest cells (CNCCs) migrate from the lateral ridges of the neural plate to the first branchial arch to form the orofacial region, including the maxilla, mandible, zygoma, trigeminal nerve, and muscles of mastication (Ito et al., 2003; Lee and Saint-Jeannet, 2011; Birgfeld and Heike, 2019). CNCCs have to migrate and undergo proliferation and differentiation to form the dentin and pulp of the teeth, connective tissues, cartilage, and bone of the head (Chai et al., 2000; Sakai and Trainor, 2009; Achilleos and Trainor, 2012). In animal studies, CNCCs populating the pharyngeal arches are characterized by expression of transcription factor Sox9 (Li et al., 2002; Cheung and Briscoe, 2003; Hong and Saint-Jeannet, 2005; Lee and Saint-Jeannet, 2011; Wu et al., 2017). In addition, Sox9 acts as a key transcription factor that is required for both early and late stages of osteogenic (Yamashiro et al., 2004; Stockl et al., 2013; Loebel et al., 2015; Rutkovskiy et al., 2016) and chondrogenic differentiation (Akiyama et al., 2002, 2005; Hattori et al., 2010; Guang et al., 2012; Henry et al., 2012; Jeon et al., 2014; Rutkovskiy et al., 2016). To initiate bone formation, CNCCs were found to form aggregated cell masses in the orofacial mesenchyme (Ito et al., 2003). In mice fetuses, Sox9 expression has been observed in the osteogenic cell compartments in the craniofacial bones between E12-E16 (Yamashiro et al., 2004). CXCL12-CXCR4 chemokine-receptor signaling drives both osteogenic and chondrogenic differentiation (Ito, 2011). CXCL12-CXCR4 signaling mediates both immature and mature murine osteoblast development (Zhu et al., 2011; Shahnazari et al., 2013). Moreover, CXCL12-CXCR4 signaling promotes Sox9-mediated chondrogenesis in synovium-derived stem cells (Wang et al., 2017), whilst blocking CXCL12-CXCR4 signaling inhibits chondrogenic differentiation *in vitro* (Guang et al., 2012). However, Sox9 knockout (KO) mice show reduced CXCR4 expression in the kidney (Reginensi et al., 2011).

CXCL12-CXCR4 signaling regulates osteoblast formation by promoting alkaline phosphatase (ALP)-activity in human (Hosogane et al., 2010; Li et al., 2017), and murine osteoblasts (Liu et al., 2013) *in vitro*. ALP-activity is often used as a marker of osteoblastic development (Stein and Lian, 1993), and ALP KO mice demonstrate defects in bone mineralization (Wennberg et al., 2000). Mesenchymal stem cells exposed to osteogenic medium with CXCL12 demonstrated higher ALP-activity, supporting a role for CXCL12 in osteogenic differentiation *in vitro* (Kortesidis et al., 2005).

Since Sox9 expression was observed in the palatal shelves in mice fetuses at E12.5, E13.5 (Watanabe et al., 2016), E14.5 (Potter and Potter, 2015; Watanabe et al., 2016), and E15 (Xu et al., 2018), migrating CNCCs are thought to contribute to the embryonic formation of the palate, named palatogenesis (Watanabe et al., 2016; Xu et al., 2018). Palatogenesis is an important event during craniofacial development of higher vertebrates (Ferguson, 1988). Our basic understanding of palatal morphogenesis comes principally from research conducted in mice. Palatogenesis occurs during the intrauterine weeks 8–12 in humans and during embryonic days E12–E15.5 in mice (Dudas et al., 2007). Palatogenesis involves the vertically downward outgrowth of the paired palatal shelves from the maxillary region, elevation above the tongue (E14.5-E15), horizontal growth and adherence, formation of the midline epithelial seam (MES), and eventually disintegration of the MES, allowing complete palatal fusion (E15.5) (Chai and Maxson, 2006; Dudas et al., 2007; Levi et al., 2011). Cranial neural crest-derived mesenchymal cells have to undergo osteogenic differentiation to form the hard palate (Xu et al., 2019). In mice, CNCCs form aggregated cell masses to initiate palatal bone formation in the palatal mesenchyme in mice between E14.5 and E16.5 (Ito et al., 2003; Oka et al., 2012). In cultured mouse palatal shelves, inhibition of osteoblast differentiation by treatment with 10 mM lithium chloride prevented palatal fusion (Meng et al., 2015).

Since CXCL12-CXCR4 expression was found to promote bone formation (Zhu et al., 2011; Shahnazari et al., 2013) and ALP-activity (Kortesidis et al., 2005), we postulate that both CXCL12 and CXCR4 are expressed within the palatal mesenchyme to facilitate palatal osteogenesis. Transcription factor Sox9 may promote CXCL12-CXCR4 signaling to initiate ALP-activity, facilitating osteoblast and chondrocyte formation within the CNCCs derived mesenchyme, starting osteogenesis in the developing orofacial region.

Midline epithelial seam disintegration is crucial for palatal fusion (Iseki, 2011). The main hypotheses underlying MES disintegration during palatal fusion involve epithelial cell migration to the oral (Aoyama et al., 2019; Logan and Benson, 2019) or nasal epithelium (Jin and Ding, 2006; Aoyama et al., 2019), epithelial-to-mesenchymal transformation (EMT) (Nawshad, 2008; Serrano et al., 2015; Nakajima et al., 2018), epithelial cell apoptosis (Lan et al., 2015; Suttorp et al., 2017), or a combination of these events (Iseki, 2011). We previously demonstrated that the MES highly expresses chemokine CXCL11, suggesting its involvement in recruiting CXCR3-positive macrophages to facilitate phagocytosis of apoptotic cells of the disintegrating MES during palatal fusion (Suttorp et al., 2017). Notably, palatal bone formation in mice starts at E14.5 (Ito et al., 2003; Oka et al., 2012), simultaneously with MES disintegration (Chai and Maxson, 2006; Dudas et al., 2007; Levi et al., 2011). Therefore, we postulate that the MES might also express other chemokines involved in other pathways, such as CXCL12, to promote maturation of immature CXCR4-positive osteoblasts and ALP-activity to start palatal osteogenesis.

Disturbed migration of CNCCs to the orofacial region can lead to craniofacial abnormalities (Wang et al., 2019). Exposure to reactive oxygen species (ROS), generated by diabetes, infections, or social poisons, including maternal smoking, alcohol consumption, and exposure to teratogens, was found to harm fetal growth and development and could even lead to craniofacial anomalies in rats (Al Ghafli et al., 2004), zebrafish (Kim et al., 2014; de Peralta et al., 2016), and humans (Simpson, 1957; Andres, 1996; Shah and Bracken, 2000; Ion and Bernal, 2015; Parks, 2015; Zadzinska et al., 2016). ROS can contribute to the etiology of congenital malformations by disrupting migration of CNCCs to the orofacial region (Sakai and Trainor, 2016). In chick embryo, ROS production generated by caffeine exposure was found to disturb CNCC migration, leading to asymmetrical microphthalmia and abnormal orbital bone development (Ma et al., 2014).

During palatogenesis, failure of elevation, horizontal growth, adherence of the palatal shelves, or disruption of MES formation and MES disintegration results in cleft palate (Nakajima et al., 2018). Palatal clefting can be caused by disturbed migration of CNCCs to the orofacial region (Wang et al., 2019). In humans, disruption of CNCC migration to the oral region can lead to cleft lip and palate (CLP) (Ito et al., 2003) and Pierre Robin sequence, a congenital craniofacial anomaly characterized by mandibular micrognathia, glossoptosis, and cleft palate (Jakobsen et al., 2006; Chen et al., 2019).

A combination of genetic and environmental factors is thought to play a role in the etiology of orofacial clefting (Spilson et al., 2001; Mossey et al., 2009; Brocardo et al., 2011). Mice lacking transcription factor AP-2a show hampered CNCC migration resulting in congenital anomalies, including cleft palate (Nottoli et al., 1998). Repression of CNCC proliferation by inhibiting the transcription factor family Zeb prevents fusion between cultured mice palatal shelves *in vitro* (Shin et al., 2018). Failure of palatal bone formation between E14.5 and E16.5 in mice is associated with cleft palate (Oka et al., 2012). Maternal smoking (Shi et al., 2008), alcohol consumption (DeRoo et al., 2008), and diabetes (Correa et al., 2008), associated with oxidative and inflammatory stress (Chen and Scholl, 2005; Albano, 2006; Kamceva et al., 2016), were found to increase the risk at babies with CLP. Gestational treatment with nicotine inhibited palatal fusion by persistence of the MES in mice fetuses (Kang and Svoboda, 2003; Ozturk et al., 2016). In pregnant CL/Fr mice, having an incidence of spontaneous CLP of 35–40% in the offspring, exposure to hypoxia at E11 doubled the incidence of CLP at E18, indicating that a combination of genetic susceptibility and oxidative stress can result in CLP(Millicovsky and Johnston, 1981).

The heme oxygenase (HO) enzyme system protects against oxidative and inflammatory stress by degrading the pro-oxidant heme, thereby generating free iron/ferritin, carbon monoxide (CO), and the antioxidants biliverdin/bilirubin (Wagener et al., 2003; Grochot-Przeczek et al., 2012; Gorlach et al., 2015). These HO-effector molecules regulate vasodilation, inhibit platelet aggregation, suppress leukocyte adhesion, and reduce pro-inflammatory cytokine release (Grochot-Przeczek et al., 2012). The HO-system consists of two functional isoforms; HO-1 has low basal levels but is strongly inducible, whereas HO-2 is constitutively expressed (Ewing and Maines, 2006). Downregulation of HO-1 (Zenclussen et al., 2003) and HO-2 (Sollwedel et al., 2005) in human placenta is associated with spontaneous abortion, pre-eclampsia, and fetal growth retardation. HO-2 KO mice demonstrated fetal growth restriction, severe malformations, craniofacial anomalies (Suttorp et al., 2017), and elevated endothelial inflammatory and angiogenic factors (Bellner et al., 2009). HO-1 KO mice demonstrated a high prenatal mortality, with survivors showing growth retardation, organ fibrosis, and inflammatory tissue damage (Kapturczak et al., 2004). Others found that HO-1 KO mice are highly susceptible to ischemia, reperfusion injury, and right ventricular infarction (Yoshida et al., 2001; Liu et al., 2005). Induction of HO-1 decreased ROS levels in obese diabetic mice (Li et al., 2008). Interestingly, HO-1 expression promoted ALP-activity in the process of differentiation of osteoblast stem cells of human (Barbagallo et al., 2010; Vanella et al., 2012; Kim et al., 2013; Guo et al., 2017), mouse (Cheng et al., 2019), and rat into osteoblasts (Gu et al., 2012). In human periodontal ligament cells, induction of HO-1 leads to upregulation of osteogenic differentiation *in vitro* (Kook et al., 2009). However, blocking of HO-activity has therapeutically been used in preterm infants since administration of SnMP, a competitive inhibitor of HO-1 and HO-2, attenuates the development of hyperbilirubinemia (Valaes et al., 1994).

We postulate that Sox9, CXCR4, and HO-1 are expressed in the ALP-activity positive osteogenic regions within the CNCCs derived mesenchyme during palatal fusion in mice. Furthermore, we hypothesize that chemokine CXCL12 is expressed by the disintegrating MES to promote the formation of an osteogenic center by CXCR4-positive osteoblasts. In addition, we expect that increased levels of oxidative and inflammatory stress in HO-2 KO mice, and further increase of these stress levels, obtained by administration of HO-1 and HO-2 activity inhibitor SnMP at E11 in wt mice disrupts the migration of CNCCs to the orofacial region, increasing the risk of cleft palate.

In the present study, expression of Sox9, CXCL12, CXCR4, and HO-1 was studied in relation to MES disintegration at E15 and palatal osteogenesis between E15 and E16 marked by ALP-activity, in wild-type (wt) mice using (immuno)histochemical staining of coronal palatal sections. Additionally, the effects of absence of HO-2 in HO-2 KO mice, or inhibition of HO-1 and HO-2 activity using SnMP from E11 in wt mice on palatal bone formation at E15 and E16, was investigated.

## Materials and Methods

### Mice Selection and Mating

Because HO-1 KO mice demonstrated severe pregnancy complications with a fetal loss rate of more than 85% (Zenclussen et al., 2011), and HO-2 mice demonstrated to be viable (Lundvig et al., 2014), the HO-2 mouse model was considered more suitable to collect fetuses for this study. Homozygote HO-2 KO mice were generated by targeted disruption of the HO-2 gene with mixed 129Sv × C57BL/6 background (Poss et al., 1995; Bellner et al., 2009). By quantitative real-time PCR, we previously confirmed the genotypes of mice by showing that HO-2 mRNA was only present in samples from wt fetuses, and not in HO-2 KO fetuses (Suttorp et al., 2017). The wt mice strain with mixed 129Sv × C57BL/6 background were used to obtain fetuses for the control group. Both the wt (*n* = 7) and HO-2 KO mice (*n* = 7) of 8 weeks old were bred and maintained in our animal facility. Female wt mice were mated with wt males, and female HO-2 KO mice were mated with HO-2 KO males. The next morning the mice were checked for the presence of a vaginal copulation plug, taken as day 0 of pregnancy (gestational/embryonic day 0; E0) (Behringer et al., 2016). Preliminary experiments (Ethical permission # RU-DEC 2009-160) demonstrated that both wt and HO-2 KO animals with mixed 129Sv × C57BL/6 background often did not carry fetuses. The hormones Folligonan (Gonadotropin serum, Intervet Nederland BV, Boxmeer, Netherlands) and Pregnyl (Human chorionic gonadotropin, NV Organon, Oss, Netherlands) were used to enhance the chance of pregnancy. At day –3 at 16.00 h Folligonan (6E in 30 μl) and at day –1 at 16.00 h Pregnyl (6E in 30 μl) was administered by intraperitoneal (i.p.) injection. At first, 1 wt mouse and two HO-2 KO mice demonstrated no plugged status. These animals were mated again 4 weeks later, and all demonstrated a plugged status the next morning.

Female CD1 mice of 12–17 weeks old (*n* = 11) were mated with male mice from the same strain at the animal facility of the animal suppliers Envigo (Venray, Netherlands). The plugged CD1 mice were transported to our animal facility and could acclimatize for at least 1 week before the start of the experiment. Absence of HO expression (both isoforms HO-1 and HO-2) was found to severely affect embryonic implantation (Zenclussen et al., 2011). SnMP can bind to the enzymes HO-1 and HO-2, but cannot be broken down by both isotypes, acting as a competitive inhibitor of the HO system (Stevenson et al., 1989). Instead of heme (iron protoporphyrin IX dichloride), SnMP (Sn mesoporphyrin IX dichloride) consists of a protoporphyrin IX ring with tin in its center (Lutton et al., 1999). SnMP was purchased from Frontier Scientific (Carnforth, United Kingdom). SnMP was freshly dissolved with Trizma base. The pH was adjusted to pH 7.6–8.0 with HCl, and further diluted till 10 mL with H_2_O. The SnMP solution was filter sterilized before administration. To be able to study palatal fusion in the absence of HO-1 and HO-2 activity (later referred to as HO-activity), pharmacological blocking in CD1 wt mice was obtained by administration of SnMP at the start of palatogenesis at E11.

### Sample Size, Housing, Ethical Permission

To detect an effect size of 0.40 (generalized estimation of the reduction in fetal body weight following HO-2 abrogation/HO-activity inhibition) with power of 0.80 and a significance level of 0.05 for the four groups (wt E15, HO-2 KO E15, wt CD1 E16, and wt CD1 SnMP E16) a total sample size of *n* = 76 fetuses for this study was calculated by the one-way ANOVA power analysis *a priori* (G^∗^Power 3.1 software) (Faul et al., 2007). This indicated that the mean sample size per group should comprise approximately 19 fetuses. We estimated that the litter size could range from 8 to 18 fetuses, suggesting that each group should contain three pregnant mice. The chance of conception was assumed to be around 70%, indicating that for each group at least five mice should be mated.

The animals of both models were housed under specific pathogen-free housing conditions with 12 h light/dark cycle and *ad libitum* access to water and powdered rodent chow.

Ethical permission for the study was obtained according to the guidelines of the Board for Animal Experiments of the Radboud University Nijmegen (Ethical permission # RU-DEC 2012-166).

### Studying Chemokine Expression by the MES in the Presence or Absence of HO-2

To be able to study chemokine expression by the MES, fetuses should be studied before the palatal shelves are completely fused. In wt mice the palatal shelves fuse between E14.5 and E15.5. In this period, the MES disintegrates, allowing the formation of mesenchymal confluence (Dudas et al., 2007). Therefore, fetuses of E15 were considered to be suitable to study chemokine expression by the disintegrating MES. In the absence of HO-2 expression chemokine expression by the MES was examined in HO-2 KO fetuses of E15 (later referred to as HO-2 KO E15). The E15 wt fetuses served as controls (later referred to as wt E15).

### Studying Palatal Fusion in the Presence or Absence of HO-Activity

To be able to study palatal clefting, fetuses should be studied beyond the time point of palatal fusion. Since the epithelium of the palatal shelves loses its capacity to fuse at E16, the absence of palatal fusion at E16 is diagnosed as palatal clefting (Dudas et al., 2007). Therefore, fetuses of E16 were considered to be suitable to study palatal fusion. In the absence of HO-activity, obtained by SnMP administration, palatal fusion was examined in wt CD1 fetuses of E16 (later referred to as wt CD1 SnMP E16). In the control group, no SnMP was administered (later referred to as wt CD1 E16). All CD1 mice were randomly assigned to the wt CD1 E16 or wt CD1 SnMP E16 group.

### Isolation of Mice Fetuses

**FIGURE 1:**
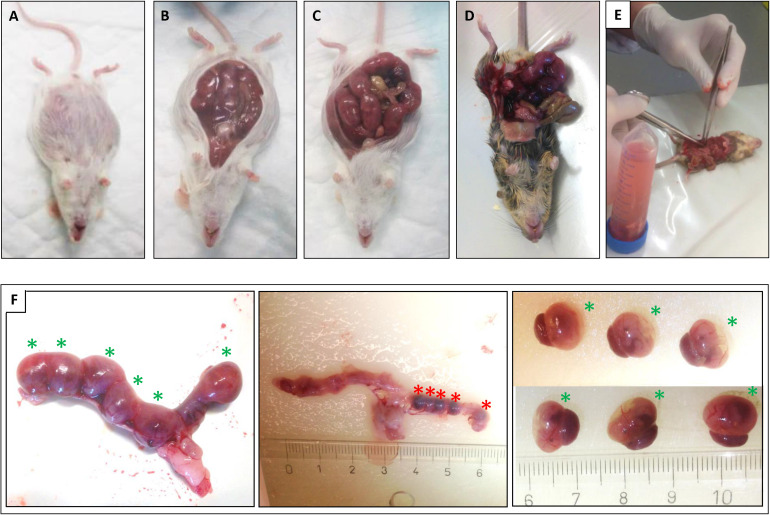
Isolation of the uteri and mice fetuses. After sacrifice of the plugged mice the uteri were isolated: **(A)** wt E15 mouse. **(B)** wt E15 mouse, **(C)** wt CD1 SnMP E16 mouse, **(D,E)** HO-2 KO E15 mouse. **(F)** Isolation of the fetuses from the uterus of a HO-2 KO E15 mouse. Left panel: Uterus containing 6 fetuses (green asterisks) in total. Middle panel: After removal of the fetuses five resorptions (red asterisks) were found (centimeter ruler). Right panel: Overview of the six isolated fetuses with placenta (green asterisks).

The plugged animals of both models were sacrificed by CO_2_/O_2_ inhalation for 10 min, and the uteri and fetuses were isolated and photographed (see Figure 1). The wt and HO-2 KO mice were sacrificed at E15. Only three out of seven plugged wt mice, and four out of seven plugged HO-2 KO mice carried fetuses. In total, 16 wt E15 fetuses and 11 HO-2 KO E15 fetuses were obtained. The CD1 wt mice, the controls, and SnMP administered mice, were sacrificed at E16. In total of six out of six plugged wt mice of the control group, and four out of five plugged wt mice of the SnMP group carried fetuses. In total, 91 wt CD1 E16 fetuses and 56 wt CD1 SnMP E16 fetuses were obtained.

### Fetal Loss Rate Calculation

For the fetus-carrying mice of both models, the fetal loss rate, the percentage of non-viable and hemorrhagic embryonic implantations to the total number of embryonic implantations (non-viable or hemorrhagic embryonic implantations + fetuses) was calculated.

### Fetal Body Weight

To study the effects of the absence of HO-2 expression on fetal growth wt E15 fetuses (*n* = 15) and HO-2 KO E15 fetuses (*n* = 4) were weighed. Unfortunately, when the isolation of the uterus was performed for the first time, we weighed the fetuses in their amniotic sacs together with their placentas. During preparation we noticed that in some cases the amniotic fluid was partially leaked away caused by a perforation of the amniotic membrane, making this method unreliable. Subsequently, it was decided to perform the weighing of the fetuses separately from their amniotic sacs and placentas. Therefore, the weight of 1 wt E15 and 7 HO-2 KO E15 fetuses was not collected. To study the effects of HO-activity inhibition on fetal growth the wt CD1 E16 fetuses (*n* = 91) and wt CD1 SnMP E16 fetuses (*n* = 56) were weighed.

### Paraffin Embedding and Section Cutting of Head Samples

Fetuses were decapitated and the head samples were fixed for 24 h in 4% paraformaldehyde and further processed for routine paraffin embedding. Serial coronal sections through the secondary palate region of 5-μm thickness were mounted on Superfrost Plus slides (Menzel-Gläser, Braunschweig, Germany). The paraffin sections were deparaffinized using Histosafe (Adamas Instrumenten B.V., Rhenen, Netherlands) and rehydrated using an alcohol range (100%–90%–80%–70%–35%–0%) for further (immuno)histochemistry.

### Palatogenesis Classification of HE-Stained Palatal Sections

Serial coronal palatal sections from the wt E15, HO-2 KO E15, wt CD1 E16 and wt CD1 SnMP E16 fetuses were routinely stained with hematoxylin and eosin (HE). Because the palate is not fused in once, the four stages according to Dudas et al. (2007) and Suttorp et al. (2017) can all be present on the same embryonic day. Therefore, the sections were classified into the four stages of palatogenesis based on the anatomy of the secondary palatal shelves: elevation, horizontal growth, midline adhesion, and fusion. Per individual fetus palatogenesis was studied on multiple sections. A minimum of five sections from five to seven head samples per group were essayed. Microscopic photographs were taken using a Carl Zeiss Imager Z.1 system (Carl Zeiss Microimaging Gmbh, Jena, Germany) with AxioVision (4.8 v) software (Zeiss, Göttingen, Germany). HE series from the wt E15 and HO-2 KO fetuses were used as reference to obtain coronal palatal sections containing the MES for (immuno)histochemical staining. HE series from the wt CD1 E16 and wt CD1 SnMP E16 fetuses were used as reference to obtain coronal palatal sections containing the middle region of the fusing palatal shelves for (immuno)histochemistry.

### Palatal Osteogenesis Identification by ALP-Activity

ALP-activity is often used as a marker of osteoblastic development (Stein and Lian, 1993). In the coronal palatal sections, increased expression of ALP activity was found to indicate enhanced differentiation of mesenchymal stem cells into osteoblasts (Rosa et al., 2003; George et al., 2006). Therefore, in this study ALP-positive stained regions were judged sites of initiated osteogenic differentiation. Paraffin embedded sections were selected from the wt E15, HO-2 KO E15, wt CD1 E16, and wt CD1 SnMP E16 fetuses. The sections were rinsed in demineralized water. Then, the sections were incubated for 60 min with TRIS buffer at 37°C, followed by incubation with Medium Alkaline Phosphatase at 37°C for half an hour. After rinsing with demineralized water, the sections were counterstained by Na-acetate buffer 0.1M pH 5.1, continued with 0.1% Methyl green in Na-acetate buffer with pH 5.1. The sections were briefly rinsed before mounting in Kaisers gelatin. Microscopic photographs of the ALP-stained palatal sections were taken.

### Immunofluorescence Staining for Sox9, CXCR4, and HO-1-Expression in the Palate

For the Sox9 immunofluorescence staining, paraffin-embedded coronal palatal sections from the wt E15 and HO-2 KO E15 fetuses containing a complete or partial MES were selected. For the CXCR4 and HO-1, and the double-staining Sox9-CXCR4 and Sox9-HO-1 paraffin embedded sections from the wt E15, HO-2 KO E15, wt CD1 E16, and wt CD1 SnMP E16 fetuses were selected. Antigens were retrieved with citrate buffer at 70°C for 10 min, following by incubation in 0.015% trypsin in PBS at 35°C for 5 min. Next, the sections were pre-incubated with 10% normal donkey serum (NDS) in phosphate-buffered saline with glycine (PBSG). First, antibodies were applied overnight (see Table 1), after rinsing, followed by the secondary antibodies (see Table 1) for 1 h. For the double staining, the second first antibodies were also applied overnight. Nuclear staining was performed with DAPI. The sections were mounted with a glycerol based mounting medium containing 1, 4-Diazobicyclo-(2,2,2-octane (DABCO). Microscopic photographs of the immunofluorescence stained sections were taken.

**TABLE 1:** First and secondary antibodies used for CXCL12, Sox9, CXCR4, and HO-1 immunohistochemical staining.

First antibody	Specificity	Concentration (μg/ml)	Source	
14-7992-81	CXCL12	10	Affymetrix eBioscience, San Diego, CA, United States	
Sc 17340	Sox9	0,33	Santa Cruz via Bio-Connect, Santa Cruz, CA, United States	
PA3-305	CXCR4	3,33	Thermo Fisher Scientific, Waltham, MA, United States	
SPA 895	HO-1	2	Enzo Life Sciences BVBA, Bruxelles, Belgium	

**Secondary antibody**	**Specificity**	**Concentration (μg/ml)**	**Color**	**Source**

101909	Donkey anti Rabbit Biotine	3,4	–	Jackson Immunoresearch Europe LTD, Ely, United Kingdom
714270	Donkey anti Goat Alexa Fluor 594	20	Red	Invitrogen Thermo Fisher Scientific, Waltham, MA, United States
A11008	Goat anti Rabbit Alexa Fluor 488	4	Green	Invitrogen Thermo Fisher Scientific, Waltham, MA, United States
1777945	Goat anti Rabbit Alexa Fluor 594	4	Red	Invitrogen Thermo Fisher Scientific, Waltham, MA, United States
				

### Immunohistochemical Staining of Palatal CXCL12 Expression

Paraffin-embedded coronal palatal sections from the wt E15 and HO-2 KO E15 fetuses, containing a full or partial MES, were selected. Endogenous peroxidase activity was quenched with 3% H_2_O_2_ in methanol for 20 min. No antigen retrieval was used. Next, the sections were pre-incubated with 10% NDS in PBSG. First antibody for CXCL12 (see Table 1) was diluted in 2% NDS in PBSG and incubated overnight at 4°C. After washing with PBSG, sections were incubated for 60 min with a biotin-labeled second antibody against host species (see Table 1) as previously described (Tan et al., 2009). Next, the sections were washed with PBSG and treated with avidin-biotin peroxidase complex (ABC) for 45 min in the dark. After extensive washing with PBSG, diaminobenzidine-peroxidase (DAB) staining was performed for 10 min for the CXCL12 staining. Finally, the nuclei were stained with Hematoxylin for 10 s and sections were rinsed for 10 min in water, dehydrated, and embedded in distyrene plasticizer xylene (DPX). Microscopic photographs of the CXCL12 stained sections were taken.

### Quantification of CXCL12 Immunoreactivity in the Palatal Epithelium

**FIGURE 2:**
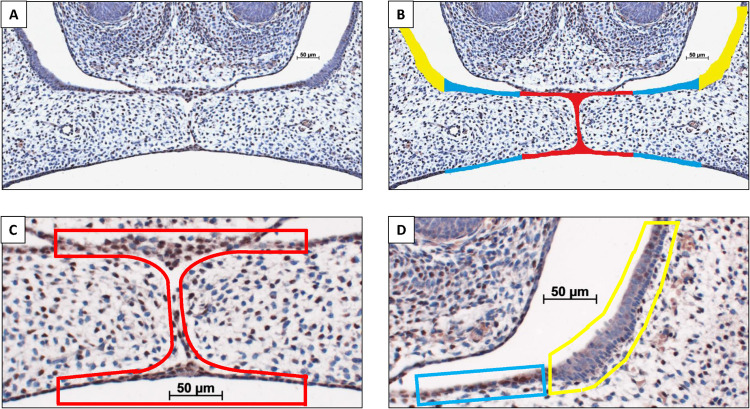
Palatal epithelium classification. **(A)** Coronal palatal section, e.g., HO-2 KO E15. **(B)** For each section the palatal epithelial layers were subdivided into three regions of interest according to morphological characteristics: (Inside) epithelium of the palatal shelves from the edge, including the MES, to half of the width of the shelves (in red); (Outside) epithelium of the lateral half of the palatal shelves (in blue); (Lateral nasal wall) epithelium of the lateral wall of the nasal cavity, this region is positioned outside the palatal shelves and served as a control region (in yellow). Immunoreactivity scoring scale: Semi-quantitative scoring of CXCL12 immunoreactivity within the epithelial regions according to the following scale: HIGH, immunoreactivity present in almost the entire epithelial region; MODERATE, immunoreactivity present only partially in the epithelial region; LOW, almost no immunoreactivity present in the epithelial region. Immunoreactivity scored for the three regions of interest in a CXCL12 immunostained section. **(C)** Inside region was scored as HIGH, in red. **(D)** Outside region was scored as MODERATE, in blue. Lateral nasal wall region was scored as LOW, in yellow.

The CXCL12 immunostained coronal palatal sections from the wt E15 and HO-2 KO E15 fetuses were examined. Within each section the epithelium of the palatal shelves was subdivided into three separate regions of interest (Inside, Outside, and Lateral nasal wall) according to morphological characteristics (epithelium region classification) as previously described (Suttorp et al., 2017). The epithelial regions per single section were semi-quantitatively scored according to the immunoreactivity scoring scale in three categories: HIGH, MODERATE and LOW (for details see Figure 2). CXCL12 immunoreactivity was evaluated by two observers, independently and blinded for the groups. The inter- and intra-examiner reliability was determined. For each individual fetus the modus of the scoring per epithelial region was calculated.

### Quantification of CXCL12-Positive Cells in the Palatal Mesenchyme

**FIGURE 3:**
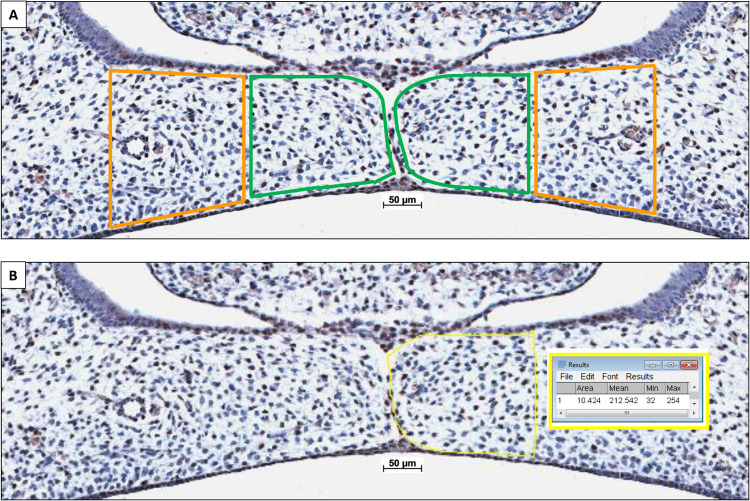
Palatal shelf cross-sectional surface measurement determining the number of CXCL12-positive immunostained cells/mm^2^ within the mesenchyme. **(A)** The individual cross-sectional surface of the inner half (in green) and outer half (in orange) of each palatal shelf was measured. **(B)** A square scale bar was drawn in the microscopic picture of 1,000×1,000 μm (mm^2^) and the total number of pixels was determined (e.g., HO-2 KO E15: 1 mm^2^ = 635,000 pixels), data not shown. The contour of the mesenchyme was drawn (yellow line). The number of pixels for this area was determines by the Fiji Image J 1.51n software (10,424 pixels). The number of CXCL12-positive stained cells within this mesenchymal area of the palatal shelves was counted (e.g., inner half contains 31 CXCL12-positive cells. The number of cells/mm^2^ was calculated (635,000/10,424 × 31 = 1888 cells/mm^2^).

Because the coronal palatal sections showed a significant variance in size of the palatal shelves, CXCL12 staining was adjusted to the surface area. The individual cross-sectional surface of the inner and outer half of each pair of shelves was measured using Fiji Image J 1.51n software (Zeiss, Göttingen, Germany). Then, the number of CXCL12-positive cells within the outline of the inner and outer half of the mesenchyme of the palatal shelves was counted (for details see Figure 3). The number of mesenchymal CXCL12-positive cells/mm^2^ per inner and outer half of the palatal mesenchyme per section was calculated. Cell counting was performed by two observers, independently and blinded for the groups. The inter- and intra-examiner reliability was determined. For each individual fetus the mean number of mesenchymal CXCL12-positive cells/mm^2^ per inner and outer half of the mesenchyme of the palatal shelves was calculated.

### Statistical Analysis

The fetal loss rate of the wt E15 and HO-2 KO E15 fetuses, and the wt CD1 E16 and wt CD1 SnMP E16 fetuses, showed a non-normal distribution as evaluated by the Kolmogorov-Smirnov test (KS-test). The differences in fetal loss rate between those groups were compared using the non-parametric Kruskal-Wallis ANOVA on ranks test and Dunn’s Multiple Comparison *post hoc* test.

The fetal body weight of the wt E15 and HO-2 KO E15 fetuses showed a normal distribution as evaluated by the KS-test. To compare differences between those groups the Independent-Samples *T*-test was performed. The fetal body weight of the wt CD1 E16 and wt CD1 SnMP E16 fetuses showed a non-normal distribution as evaluated by the KS-test. The non-parametric Mann-Whitney test was used to compare differences between both groups.

The CXCL12 immunoreactivity in the three regions of interest of the palatal epithelium of the wt E15 and HO-2 KO E15 fetuses was semi-quantitatively scored and analyzed using the non-parametric Kruskal-Wallis ANOVA on ranks test and Dunn’s Multiple Comparison *post hoc* test to compare differences between both groups.

The number of CXCL12-positive cells/mm^2^ within the outline of the inner and outer half of the palatal mesenchyme of the wt E15 and HO-2 KO E15 fetuses was quantitatively scored and showed a normal distribution as evaluated by the KS-test. The data was analyzed using the ANOVA and Tukey’s multiple comparison *post hoc* test to compare differences between the inner and outer half of the palatal mesenchyme of both groups.

To determine the inter- and intra-examiner reliability for the semi-quantitative data of the CXCL12 immunoreactivity in the epithelium, the Cohen’s kappa coefficient was calculated. Acceptable scores >0.80 were obtained for the semi-quantitatively scoring.

To determine the inter- and intra-examiner reliability, the coefficient of determination (*R*^2^) was calculated by the square of the Pearson correlation coefficient for the quantitative data of the CXCL12-positive cells/mm^2^ in the palatal mesenchyme. Acceptable scores >0.80 were obtained for the counting.

Differences were considered to be significant if *p* < 0.05. All statistical analyses were performed using Graphpad Prism 5.03 software (GraphPad Software, San Diego, CA, United States).

## Results

### MES Disintegration Despite HO-2 Abrogation and Palatal Fusion Despite HO-Activity Inhibition From E11

**FIGURE 4:**
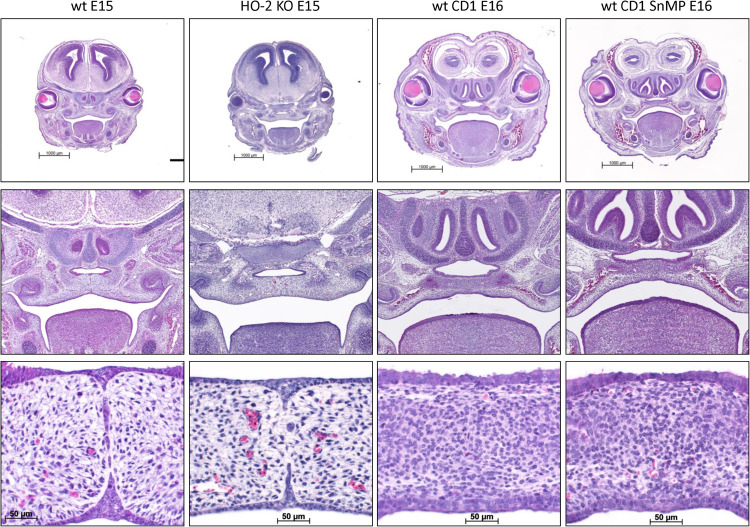
MES disintegration despite HO-2 abrogation and palatal fusion despite HO-activity inhibition from E11. In the HE-stained coronal palatal sections of the wt E15 and HO-2 KO E15 fetuses, the palatal shelves were attached and disintegration of the MES was found. In the HE-stained coronal palatal sections from wt CD1 E16 and wt CD1 SnMP E16 fetuses, the palatal shelves were fused. Representative palatal section per group was shown.

In the HE-stained coronal palatal sections of the wt E15 and HO-2 KO E15 fetuses, the tips of the palatal shelves were attached and disintegration of the MES was found. In the HE-stained sections from the wt CD1 E16 and wt CD1 SnMP E16 fetuses, the palatal shelves were fused. Representative palatal section per group was shown in Figure 4.

### Palatal Osteogenesis During MES Disintegration Is Normal Despite HO-Activity Inhibition

**FIGURE 5:**
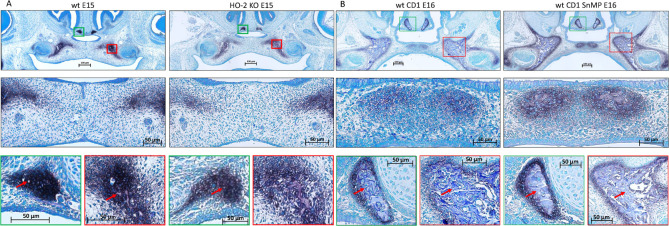
Palatal osteogenesis during MES disintegration: **(A)** Upper panel: Histochemical stained coronal palatal section for ALP-activity, representative for the wt E15 and HO-2 KO E15 fetuses. Middle panel: Magnification of the central part of the fusing palate including the MES. The palatal mesenchymal cells demonstrated positive staining for ALP-activity. Green panel: Magnification of the nasal septum. A cluster of ALP-activity positive-stained mesenchymal cells was found in the region lateral from the nasal septum including some small bone matrix depositions (red arrow). Red panel: Magnification of the lateral part of the palatal shelve. A cluster of ALP-positive stained mesenchymal cells was found including small bone matrix depositions (red arrow). **(B)** Upper panel: Histochemical stained sections through the maxilla for ALP-activity, representative for the wt CD1 E16 and wt CD1 SnMP E16 fetuses. Middle panel: Magnification of the central part of the fusing palate. A cluster of ALP-positive stained mesenchymal cells including bone matrix depositions was found at the former location of the MES and was regarded as an osteogenic center. Green panel: Magnification of the nasal septum. A cluster of ALP-positive-stained mesenchymal cells surrounding a large area of bone matrix deposition (red arrow) was found. Red panel: Magnification of the lateral part of the palatal shelve. A cluster of ALP-positive-stained mesenchymal cells in the lateral part of the fusing palate surrounding a large area of bone matrix deposition (red arrow) was found.

In the coronal palatal sections of the wt E15 and HO-2 KO E15 fetuses, clusters of ALP-positive-stained mesenchymal cells including small bone matrix depositions were found in the regions lateral of the fusing palatal shelves and lateral near the nasal septum. No disruption of ALP expression due to HO-2 abrogation was found. In the coronal palatal sections of the wt CD1 E16 and wt CD1 SnMP E16 fetuses, clusters of ALP-positive-stained mesenchymal cells surrounding large areas of bone depositions were found in both the center and lateral regions of the palatal shelves, and lateral near the nasal septum. Because of their ALP-activity, and their location around bone depositions, these ALP-positive clustered cells were regarded as osteoblast progenitors and osteoblasts. No disruption of ALP activity due to HO-activity inhibition was found. Representative section per group are shown in Figure 5. The clusters of ALP-positive-stained mesenchymal cells of the fusing palate were considered as palatal osteogenic centers and determined as region of interest for studying CXCL12, CXCR4, and Sox9 expression.

### Sox9 Expressing Cells Present in the MES and Palatal Osteogenic Centers

**FIGURE 6:**
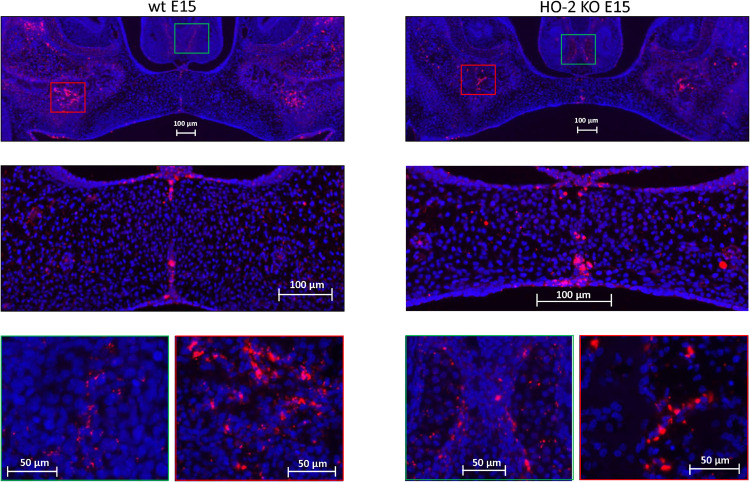
Sox9 expressing cells in the MES and the palatal osteogenic centers. Upper panels: Fluorescent immunohistochemical-stained coronal palatal sections for Sox9-activity (red), representative for the wt E15 fetuses and HO-2 KO E15 fetuses. Middle panels: Strong Sox9 expressing cells (red) were found in the remnants of the MES. Green panels: Sox9 expressing mesenchymal cells were present in the osteogenic centers at the oral side of the forming nasal septum, and also in the cartilage of the nasal septum. Red panels: The osteogenic centers in the lateral parts of the fusing palate demonstrated clusters of strong Sox9 expressing cells.

In both wt E15 and HO-2 KO E15 fetuses, Sox9 high-expressing cells were found in the disintegrating MES. Moreover, clusters of Sox9-positive mesenchymal cells were found in the osteogenic centers in the lateral parts of the fusing palate and at the oral side of the forming nasal septum. Regarding their clustered arrangement in the osteogenic centers it was assumed that these Sox9-positive cells are predominantly osteoblast progenitors. Furthermore, Sox9 expressing mesenchymal cells were present in the cartilage of the nasal septum, and are likely chondroblasts because of their specific position (see Figure 6). The non-specific autofluorescence is shown in Supplementary Figure 1A.

### CXCR4 Expressing Cells in the Palatal Osteogenic Centers

**FIGURE 7:**
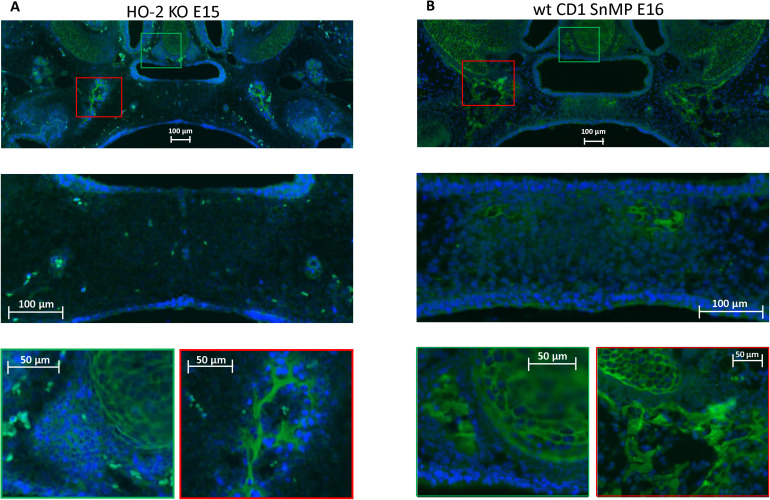
CXCR4 expressing cells in the palatal osteogenic centers. **(A)** Upper panel: Fluorescent immunohistochemical-stained coronal palatal section for CXCR4 expression (green), representative for the wt E15 and HO-2 KO E15, e.g., HO-2 KO. Middle panel: Within the disintegrating MES almost no CXCR4 expressing cells were found. Green panel: Near the forming nasal septum clusters of strong CXCR4 expressing cells were found. Also within the cartilage of the forming nasal septum CXCR4 expressing cells were present. Red panel: The osteogenic centers in the lateral parts of the fusing palate demonstrated clusters of strong CXCR4 expressing cells. **(B)** Upper panel: Fluorescent immunohistochemical stained coronal palatal section for CXCR4 expression (green), representative for the wt CD1 E16 and wt CD1 SnMP E16 fetuses, e.g., wt CD1 SnMP E16. Middle panel: in the osteogenic centers in the central part of the fusing palate clusters of CXCR4-positive-stained mesenchymal cells were found. Green panel: Near the forming nasal septum clusters of strong CXCR4 expressing cells were found. Also within the cartilage of the forming nasal septum CXCR4 expressing cells were present. Red panel: The osteogenic centers in the lateral parts of the fusing palate demonstrated clusters of strong CXCR4 expressing cells.

In both wt E15 and HO-2 KO E15 fetuses, clusters of CXCR4-positive mesenchymal cells were found in the osteogenic centers in the lateral parts of the fusing palate. No CXCR4 expression was found within the disintegrating MES. In the wt CD1 E16 and wt CD1 SnMP E16 fetuses, CXCR4-positive stained cells in the osteogenic centers in both the central and lateral parts of the forming palate were found. Furthermore, at the oral side of the forming nasal septum CXCR4 expressing cells were found at E15 and E16 (see Figure 7). Regarding their clustered arrangement in the osteogenic centers, these CXCR4-positive mesenchymal cells were supposed to be primarily osteoblasts. In the cartilage of the nasal septum, the observed CXCR4-positive cells were considered to be generally chondroblasts. The non-specific staining of the second antibody for CXCR4 is shown in Supplementary Figure 1B.

### Most CXCR4-Positive Cells in the Palatal Osteogenic Centers Are Not Positive for Sox9

**FIGURE 8:**
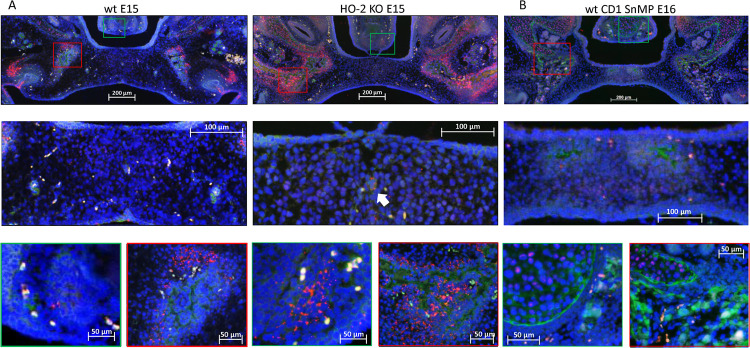
Most CXCR4-positive cells in the palatal osteogenic centers are not positive for Sox9. **(A)** Upper panel: Fluorescent immunohistochemical double-stained coronal palatal section for Sox9 (red) with CXCR4 (green), representative for the wt E15 and HO-2 KO E15 fetuses. Middle panel: Near the disintegrating MES only a few Sox9-CXCR4 double-positive stained cells (white arrow) were found. Green panel: In the osteogenic centers near the forming nasal septum clusters of Sox9 expressing cells together with CXCR4 expressing cells. Red panel: The osteogenic centers in the lateral parts of the fusing palate demonstrated clusters of Sox9 expressing cells near CXCR4 expressing cells. **(B)** Upper panel: Fluorescent histochemical double-stained coronal palatal section for Sox9 (red) with CXCR4 (green), representative for the wt CD1 E16 and wt CD1 SnMP E16 fetuses, e.g., wt CD1 SnMP E16. Middle panel: In the osteogenic centers in the central part of the fusing palate, almost no Sox9-CXCR4 double-positive stained cells were found. Green panel: Near the forming nasal septum clusters of CXCR4 expressing cells were found near CXCR4 positive cells. Also in the cartilage of the nasal septum CXCR4 positive cells were found close to Sox9 positive cells. Red panel: The osteogenic centers in the lateral parts of the fusing palate demonstrated clusters of Sox9 expressing cells near CXCR4 expressing cells.

In both wt and HO-2 KO E15 fetuses, clusters of Sox9 expressing cells surrounding CXCR4-positive cells in the osteogenic centers in the lateral parts of the forming palate were found (see Figure 8). Only a few Sox9-CXCR4 double-positive stained cells were found near the remnants of the MES. In the wt CD1 E16 and wt CD1 SnMP E16 fetuses, almost no Sox9-CXCR4 double-positive stained cells were found in the osteogenic centers in the central part of the forming palate. In general, less Sox9 expressing cells were found in the palatal mesenchyme in the wt CD1 E16 and wt CD1 SnMP E16 fetuses compared to the wt E15 and HO-2 KO E15 fetuses. In the osteogenic centers, Sox9 expressing cells were surrounding CXCR4-positive cells. At the oral side of the forming nasal septum CXCR4 expressing cells near Sox9 expressing cells were found at E15 and E16. The observation that Sox9-positive cells were located close to CXCR4-positive cells surrounding the osteogenic centers supports the suggestion that these Sox9-positive cells could be osteoblast progenitors to maintain the osteoblast pool to drive osteogenesis.

### HO-1 Expressing Cells in the Palatal Osteogenic Centers

**FIGURE 9:**
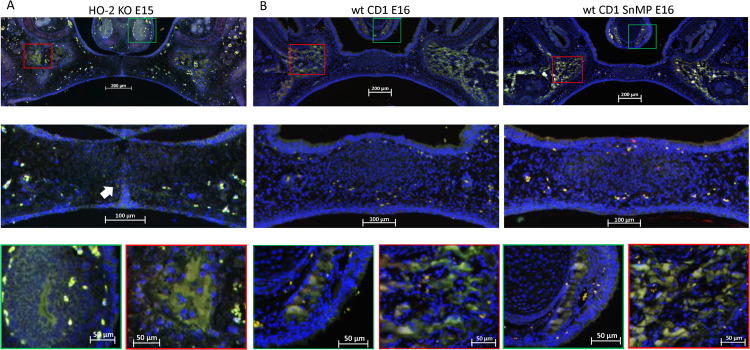
HO-1 expressing cells in the palatal osteogenic centers. **(A)** Upper panel: Fluorescent histochemical stained coronal palatal section for HO-1 expression (green), representative for the wt E15 and HO-2 KO E15 fetuses, e.g., HO-2 KO E15. Middle panel: In the mesenchyme near the disintegrating MES some HO-1 expressing cells (white arrow) were found. Green panel: Near the forming nasal septum clusters of strong HO-1 expressing cells were found. Red panel: The osteogenic centers in the lateral parts of the fusing palate demonstrated clusters of strong HO-1 expressing cells. **(B)** Upper panel: Fluorescent histochemical-stained coronal palatal section for HO-1 expression (green), representative for the wt CD1 E16 and wt CD1 SnMP E16 fetuses. Middle panel: In the central part of the fusing palate some HO-1 expressing cells were found. Green panel: Near the forming nasal septum clusters of strong HO-1 expressing cells were found. Red panel: The osteogenic centers in the lateral parts of the fusing palate demonstrated clusters of strong HO-1 expressing cells.

In both wt E15 and HO-2 KO E15 fetuses, HO-1-positive-stained cells were found in the osteogenic centers in the central part but mainly in the osteogenic centers in the lateral parts of the forming palate. In the wt CD1 E16 and wt CD1 SnMP E16 fetuses, a few HO-1-positive stained cells were found in the osteogenic centers in the central part, but clusters of HO-1-positive stained cells were dominantly found in the osteogenic centers in the lateral parts of the forming palate. At the oral side of the forming nasal septum, HO-1 expressing cells were found at E15 and E16 (see Figure 9). Since many HO-1-positive cells were found in the osteogenic centers, they were considered to be predominantly osteoblasts.

### Most HO-1-Positive Cells in the Palatal Osteogenic Centers Are Not Positive for Sox9

**FIGURE 10:**
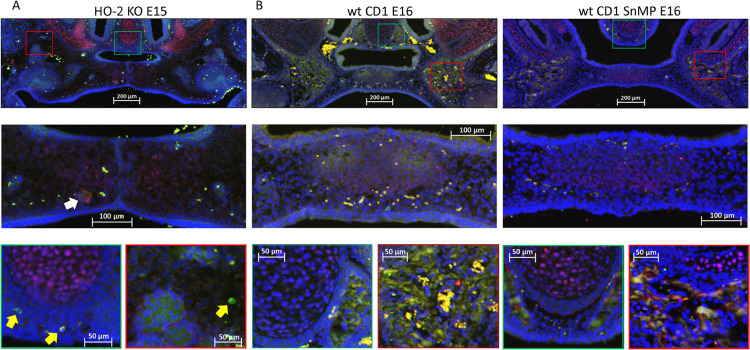
Most HO-1 positive cells in the palatal osteogenic centers are not positive for Sox9. **(A)** Upper panel: Fluorescent histochemical double-stained coronal palatal section for Sox9 (red) with HO-1 (green), representative for the wt E15 and HO-2 KO E15 fetuses, e.g., HO-2 KO E15. Middle panel: Near the disintegrating MES Sox9-HO-1 double-positive-stained cells (white arrow) were sporadically found. Green panel: In the osteogenic centers near the forming nasal septum clusters some HO-1 expressing cells were found. Furthermore, some solitary HO-1 expressing cells (yellow arrow) were found. In the cartilage of the nasal septum, multiple Sox9 expressing cells were found. Red panel: The osteogenic centers in the lateral parts of the fusing palate demonstrated clusters of HO-1 expressing cells, and solitary strong HO-1 expressing cells (yellow arrows) in the mesenchyme. **(B)** Upper panel: Fluorescent histochemical double-stained coronal palatal section for Sox9 (red) with HO-1 (green), representative for the wt CD1 E16 and wt CD1 SnMP E16 fetuses. Middle panel: In the fusing palate clusters of HO-1, expressing cells were found within the palatal osteogenic centers. Green panel: Near the forming nasal septum clusters of HO-1 expressing cells were found. In the cartilage of the nasal septum multiple Sox9 expressing cells were found. Red panel: The osteogenic centers in the lateral parts of the fusing palate demonstrated clusters of HO-1 expressing cells.

In both wt and HO-2 KO E15 fetuses, only a few Sox9-HO-1 double-positive stained cells were found near the remnants of the MES (see Figure 10). In the wt CD1 E16 and wt CD1 SnMP E16 fetuses, HO-1 expressing cells were found in the osteogenic centers in the central part of the forming palate, but almost no Sox9-HO-1 double-positive stained cells were found. At the oral side of the forming nasal septum, clusters of HO-1 expressing cells were present (see Figure 10). The examination that Sox9-positive cells surrounded the osteogenic centers containing multiple HO-1-positive cells possibly means that osteoblast progenitors are not HO-1 positive.

### CXCL12 Expression Present in the MES and Palatal Osteogenic Centers

**FIGURE 11:**
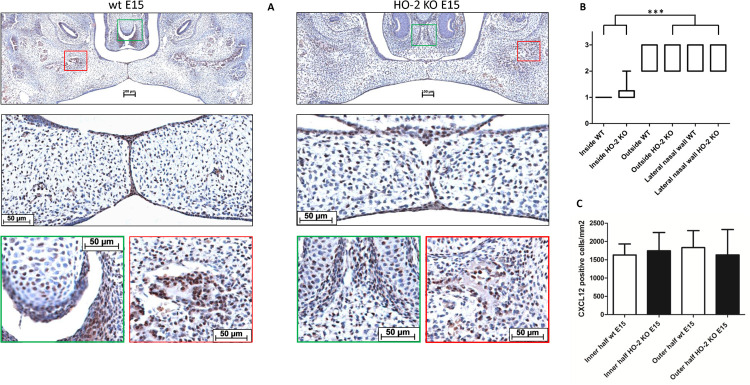
CXCL12 expression in the MES and palatal osteogenic centers. **(A)** Upper panel: Coronal palatal section stained for CXCL12 expression, representative for the wt and HO-2 KO fetuses. Middle panel: Magnification of the central part of the fusing palate including the MES. The MES demonstrated strong CXCL12 expression. CXCL12-positive cells were also found in the palatal mesenchyme. Green panel: The osteogenic centers at the lateral/oral side of the nasal septum demonstrated clusters of strong CXCL12 expressing cells. Also in the cartilage of the forming nasal septum CXCL12 expressing cells were present. Red panel: The osteogenic centers in the lateral parts of the fusing palate demonstrated clusters of strong CXCL12 expressing cells. **(B)** Box-and-whisker plot with 10–90 percentiles of semi-quantitative assessment of the CXCL12 expression in the palatal epithelial layers (scoring scale in three categories: 1 = HIGH, 2 = MODERATE and 3 = LOW) compared for the different regions in sections from wt E15 (*n* = 7) and HO-2 KO E15 fetuses (*n* = 10), ^∗∗∗^ = *p* < 0.001. **(C)** Bar chart of the number of mesenchymal CXCL12-positive cells cells/mm^2^ compared for the wt E15 (*n* = 7) and HO-2 KO E15 fetuses (*n* = 10) between the outline of the inner and outer half of the mesenchyme. Data are shown as mean ± SD. No statistically significant differences in CXCL12 expression were found (*p* = 0.85).

Chemokine CXCL12 was significantly higher expressed in the inside epithelial region including the MES (Inside) of the wt E15 and HO-2 KO E15 fetuses compared to the other epithelial layers of the fusing palatal shelves (Outside), and the lateral epithelium of the nasal cavity (Lateral nasal wall) (*p* < 0.001) (see Figures 11A,B).

CXCL12 expressing cells were also observed in the mesenchyme of the palatal shelves, but the number of CXCL12-positive cells/mm^2^ in palatal section showed no significant difference between the wt E15 and HO-2 KO E15 fetuses, and between the inner half and outer half (*p* = 0.85) (see Figure 11C). Based on their uniformly distribution, a majority of these CXCL12-positive cells in the palatal mesenchymal were considered to be fibroblasts. Furthermore, the osteogenic centers in the lateral parts of the fusing palate demonstrated clusters of strong CXCL12 expressing cells. Considering their clustered arrangement in the osteogenic centers, these CXCL12-positive cells were thought to be osteoblast progenitors or osteoblasts. Also within the cartilage of the forming nasal septum CXCL12 expressing cells were present, and these cells were regarded as chondroblast because of their specific location (see Figure 11A).

### Fetal Resorption Independent of HO-2 Expression and HO-Activity

**FIGURE 12:**
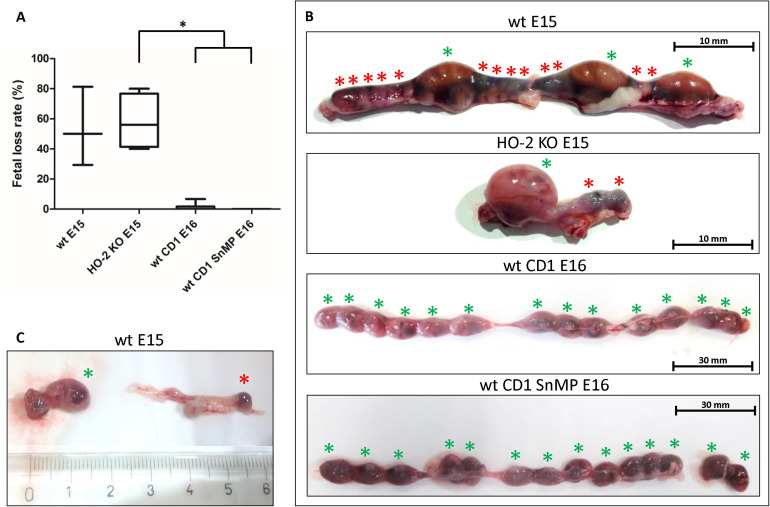
Fetal resorption independent of HO-2 KO expression and HO-activity. **(A)** Box-and-whisker plot with 10–90 percentiles of quantitative assessment of the fetal loss ratio in the wt (*n* = 3) and HO-2 KO pregnant mice (*n* = 4) at E15, and wt CD1 mice (*n* = 6) and wt CD1 SnMP mice (*n* = 4) at E16, ^∗^*p* < 0.05. **(B)** The uteri were photographed, the green asterisk indicated a fetus, the red asterisk indicated a fetal resorption. Representative uterus per group was shown. **(C)** Isolated fetus with placenta and isolated fetal resorption from the uterus.

Approximately half of the wt E15 and HO-2 KO E15 fetuses were resorbed. Contrarily, in the wt CD1 E16 and wt CD1 SnMP E16 fetuses, the number of fetal resorptions was low (less than 7%). Higher fetal loss rate was found in the HO-2 KO E15 group compared to both the wt CD1 E16 and wt CD1 SnMP groups (*p* < 0.05) (see Figure 12). Disruption of HO-2 expression and HO-activity inhibition both did not result in increased fetal loss (*p* > 0.05).

### Fetal Body Weight Is Decreased by Absence of HO-2 Expression but Increased by HO-Activity Inhibition From E11

**FIGURE 13:**
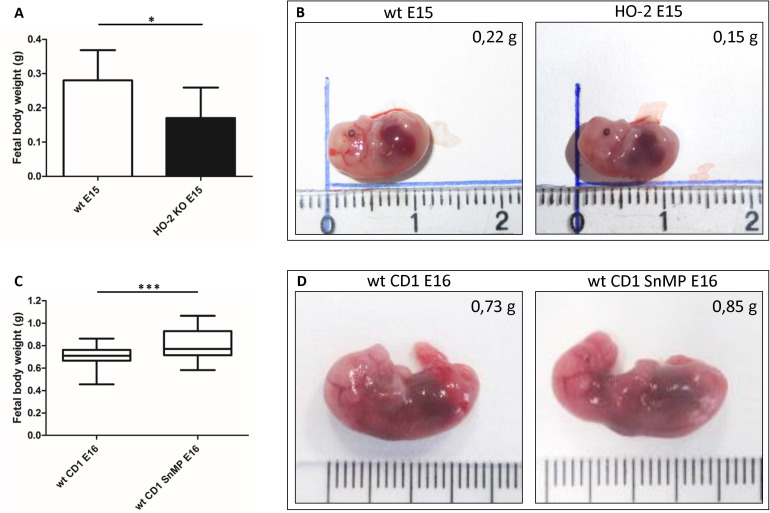
Fetal body weight decreases by disruption of HO-2, but increases by HO-activity inhibition from E11. **(A)** Bar chart of the fetal body weight of the wt E15 fetuses (*n* = 15) and HO-2 KO E15 fetuses (*n* = 4), ^∗^*p* < 0.05. Data are shown as mean ± SD. **(B)** Representative fetus per group was shown (centimeter ruler). **(C)** Box-and-whisker plot with 10–90 percentiles of quantitative assessment of the body weight of the wt CD1 E16 fetuses (*n* = 91) and wt CD1 SnMP E16 fetuses (*n* = 56), ^∗∗∗^*p* < 0.001. **(D)** Representative fetus per group was shown (centimeter ruler).

The HO-2 KO E15 fetuses demonstrated a reduced fetal body weight compared to the wt E15 fetuses (*p* = 0.0416) (Figures 13A,B). On the other hand, in CD1 fetuses of E16, inhibition of the HO-activity by SnMP from E11 increased the fetal body weight (*p* < 0.0001) (Figures 13C,D). A representative fetus per group is shown in Figures 13B,D.

## Discussion

We found clusters of Sox9 expressing cells surrounding CXCR4-positive cells in the ALP-positive osteogenic centers of the palatal shelves at E15 and E16 in wt mice. However, most Sox9 positive cells in the palate were not positive for CXCR4 expression. Furthermore, we showed that chemokine CXCL12, the ligand of receptor CXCR4, was overexpressed within these palatal osteogenic centers. Sox9, CXCR4, and CXCL12 expressing cells were also observed within the cartilage of developing nasal septum, suggesting their involvement in chondrogenesis. Several *in vitro* and *in vivo* studies on the role of CXCL12-CXCR4 signaling in relation to bone formation have been performed (Gilbert et al., 2019). CXCL12 expression in non-differentiated mesenchymal stem cells in bone was demonstrated, but toward the end of osteogenic differentiation CXCL12 was downregulated (Ito, 2011). Deletion of CXCL12 in osteoprogenitor cells resulted in decreased bone mass in the femur bone in mice (Tzeng et al., 2018). CXCL12 antibody administration inhibited new bone formation during the repair of femoral bone graft in mice (Kitaori et al., 2009). CXCR4 KO fetuses of E18.5 were smaller and showed deficient bone marrow development compared to the controls (Ma et al., 1998). CXCR4-deficient bone marrow-derived mesenchymal stromal stem cells from mice exhibited impaired osteogenic differentiation in response to BMP2 stimulation *in vitro* (Guang et al., 2013). Primary cultures for osteoblastic cells derived from CXCR4 KO mice showed decreased proliferation and impaired osteoblast differentiation in response to BMP2 or BMP6 stimulation *in vitro* (Zhu et al., 2011). Additionally, disruption of CXCR4 receptor in mouse hematopoietic stem cells led to increased endogenous ROS production (Zhang et al., 2016). Since our results showed that Sox9 and CXCR4 do not have overlapping expression, we suggest that Sox9-positive cells are osteoblast progenitors maintaining the osteoblast pool to drive osteogenesis, and that both CXCL12 and CXCR4 are later expressed by the mature osteoblasts. Furthermore, we propose that Sox9 expression and CXCL12-CXCR4 signaling in the cartilage of the nasal septum develop chondroblast formation. Since CXCL12-CXCR4 signaling promotes osteoblast formation, it is suggesting that CXCL12 expression by the disintegrating MES is a major initiator of palatal osteogenesis.

Studies on CXCL12-CXCR4 signaling in the developing palate are scarce. We provided novel evidence that chemokine CXCL12, the ligand of receptor CXCR4, was overexpressed by the epithelial cells of the MES, possibly to activate osteoblasts progenitors to facilitate palatal bone formation. In human palatal sections, CXCR4 expression was observed within the MES (Jakobsen et al., 2009). On the contrary, in our study we did not observe CXCR4 overexpression within the disintegrating MES. We could not find other studies that demonstrated CXCR4 expression in the MES. It is suggesting that CXCL12 expression by the disintegrating MES is a major initiator of palatal osteogenesis. Besides chemokine CXCL12, the disintegrating MES demonstrated strong expression of Sox9. Although Sox9 expression is demonstrated in the developing palatal shelves in mice fetuses (Potter and Potter, 2015; Watanabe et al., 2016; Xu et al., 2018), to our knowledge we are the first who demonstrated Sox9 expression specifically in the disintegrating MES. Sox9 signaling showed to be essential in the EMT mechanism in non-small-cell lung carcinoma cell (Zhang et al., 2017; Huang et al., 2019), mouse embryonic mammary cells (Kogata et al., 2018), mouse gastric cancer cells (Li et al., 2018), human colorectal cancer cells (Choi et al., 2017), human hepatocellular carcinoma cells (Kawai et al., 2016), thyroid cancer cells (Huang and Guo, 2017), and avian neural crest cells (Sakai et al., 2006) *in vitro*. Therefore, it’s tempting to speculate that Sox9 expression near the MES is involved in EMT processes, turning the basal epithelial layer of the MES into mesenchyme during palatal fusion.

**FIGURE 14:**
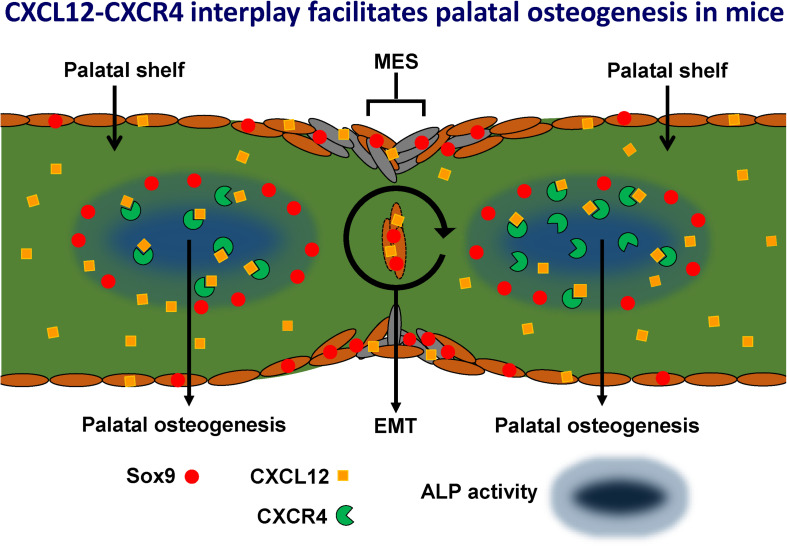
Hypothetical model: CXCL12-CXCR4 interplay facilitates palatal osteogenesis. We propose that the CXCL12-CXCR4 interplay facilitates osteogenesis during palatal fusion in mice. Expression of transcription factor Sox9 in osteoblast progenitors was thought to initiate differentiation into osteoblasts by promoting CXCL12-CXCR4 signaling. CXCL12 expression in the midline epithelial seam could promote maturation of immature CXCR4-positive osteoblast. Sox9 progenitors maintain the CXCR4-positive osteoblast pool to drive osteogenesis. Furthermore, Sox9 expression found in the MES, possibly regulates MES disintegration by the process of epithelial-to-mesenchymal transformation (EMT).

Our novel hypothetical model (Figure 14) proposes that CXCL12-CXCR4 signaling facilitates osteogenesis during palatal fusion in mice. Expression of Sox9 in osteoblast progenitors was thought to initiate osteogenic differentiation by promoting CXCL12-CXCR4 signaling. CXCL12 expression by the MES and osteogenic centers in the fusing palatal shelves could promote maturation of immature CXCR4-positive osteoblasts. Sox9 progenitors thus seem important to maintain the CXCR4-positive osteoblast pool to drive osteogenesis. Furthermore, Sox9 expression found in the MES may in addition regulate MES disintegration by the process of EMT.

Only a few Sox9-positive cells in the palatal mesenchyme were also positive for HO-1. Studies on the role of HO-activity on embryonic craniofacial development in mice are limited (Suttorp et al., 2017). As far as we know, this is the first study on the effects of inhibition of HO-1 and HO-2 by SnMP administration during palatogenesis. Adult HO-1 KO mice demonstrate growth retardation (Suliman et al., 2017), but HO-2 KO mice were found to be morphologically indistinguishable from wt mice (Poss et al., 1995). In rat, HO-1 inhibition by zinc deuteroporphyrin IX 2,4 bis glycol resulted in a significant decrease in pup size (Kreiser et al., 2002). This study showed that neither adhesion of the palatal shelves nor Sox9 and CXCL12 expression by the MES was affected by HO-2 KO disruption. We found that pharmacologically blocking of HO-activity by SnMP did not disturb palatal fusion or expression of Sox9, CXCR4, HO-1, and ALP-activity in the osteogenic regions.

Besides the absence of HO-activity, we did not further disturb the pregnancy by additional oxidative and inflammatory stress. Interestingly, HO-1 expression, but not HO-2, was found to rescue mesenchymal stem cells from H_2_O_2_-induced cell death *in vitro* (Cremers et al., 2014). It is likely that the role of HO is underestimated when its function is studied only during normal physiological conditions. For future experiments we consider to combine HO-activity inhibition with heme administration in different dose to study the effects of higher oxidative and inflammatory stress levels at palatal fusion. More research is needed to elucidate the relation between HO-activity and palatogenesis during stress-induced pathological pregnancy.

In our study, HO-2 abrogation demonstrated no increased fetal loss since both the HO-2 KO mice and wt mice showed a high fetal loss rate. Others found that HO-2 KO mice were fertile (Poss et al., 1995; Lundvig et al., 2014), although, no information was provided about the litter size. In another study, an increased abortion rate in mice was experimentally generated by lipopolysaccharide (LPS) injection at E18 in a dose-dependent fashion (Toyama et al., 2015). A limitation of the present study was the relatively small number of wt E15 and HO-2 KO E15 fetuses. Although we tried to enhance the fertility by administration of the hormones Folligonan and Pregnyl before mating, only three out of seven plugged wt mice, and four out of seven plugged HO-2 KO mice carried fetuses. It is suggested that the low fertility and high fetal loss rate in our mice with mixed 129Sv × C57BL/6 background possibly have dominated the effects of HO-2 abrogation in this study. However, our data could not provide evidence for this statement. The low fertility could possibly be explained by the excessive inbreeding within the colony. For future studies this problem could be solved by breeding homozygous HO-2 knockouts with hybrid 129Sv × C57BL/6 wt mice. By breeding the resulting heterozygotes, wt and homozygous HO-2 KO mice would then be obtained. HO-activity inhibition from E11 was not found to increase the fetal loss rate compared to the controls. HO-activity inhibition by administration of zinc mesoporphyrin early in pregnancy in mice between E0-E6 was also found to increase the abortion rate (Sollwedel et al., 2005; Schumacher et al., 2012). We demonstrated that HO-2 KO fetuses showed a reduced fetal body weight, but, interestingly, pharmacological blocking of HO-activity by SnMP administration at E11 resulted into a higher fetal body weight. This suggests that during implantation HO-activity promotes fetal growth, whereas HO-activity at later moments (E11–E16) inhibits embryonal growth. In fact, others showed that adult HO-2 KO mice were obese, induced by disrupted metabolic homeostasis, caused by insulin resistance and elevated blood pressure (Cao et al., 2012). It would then be interesting to investigate whether induction of HO-1 could indeed counteract this obesity. However, no difference in phenotype was found between HO-2 KO and wt mice by others (Qu et al., 2005). We hypothesize that HO-activity is especially essential during implantation and early embryonic development, but stress-level-dependent in the later stages of fetal development.

In conclusion, our results support the hypothesis that CXCL12-CXCR4 interplay facilitates palatal osteogenesis during palatal fusion in mice. To the best of our knowledge, this is the first study demonstrating Sox9 and CXCL12 expression in the disintegrating MES and other palatal osteogenic centers. Neither CXCL12 expression during the MES disintegration nor palatal fusion was affected by HO-2 abrogation or inhibition of HO-activity. Further research is needed to unravel the role of cytoprotective HO-activity in the presence of additional oxidative and inflammatory stresses in relation to the development of craniofacial malformations, including palatal clefting.

## Data Availability Statement

The raw data supporting the conclusions of this article will be made available by the authors, without undue reservation.

## Ethics Statement

The animal study was reviewed and approved by Board for Animal Experiments of the Radboud University Nijmegen (Ethical permission # RU-DEC 2012-166).

## Author Contributions

NV and CS designed the experiments, performed the experiments, analyzed the data, and wrote the manuscript. RER performed the experiments. RFR provided the mice. MH performed the experiments. AK-J wrote the manuscript and supervised the research. FW designed the experiments, analyzed the data, wrote the manuscript, and supervised research. All authors contributed to the article and approved the submitted version.

## Conflict of Interest

The authors declare that the research was conducted in the absence of any commercial or financial relationships that could be construed as a potential conflict of interest.
