# Supplementary figures and images for: CXCL12-CXCR4 Interplay Facilitates Palatal Osteogenesis in Mice
Source: Front Cell Dev Biol. 2020 Aug 21;8:771. doi: 10.3389/fcell.2020.00771 (PMC7471603; doi:10.3389/fcell.2020.00771)

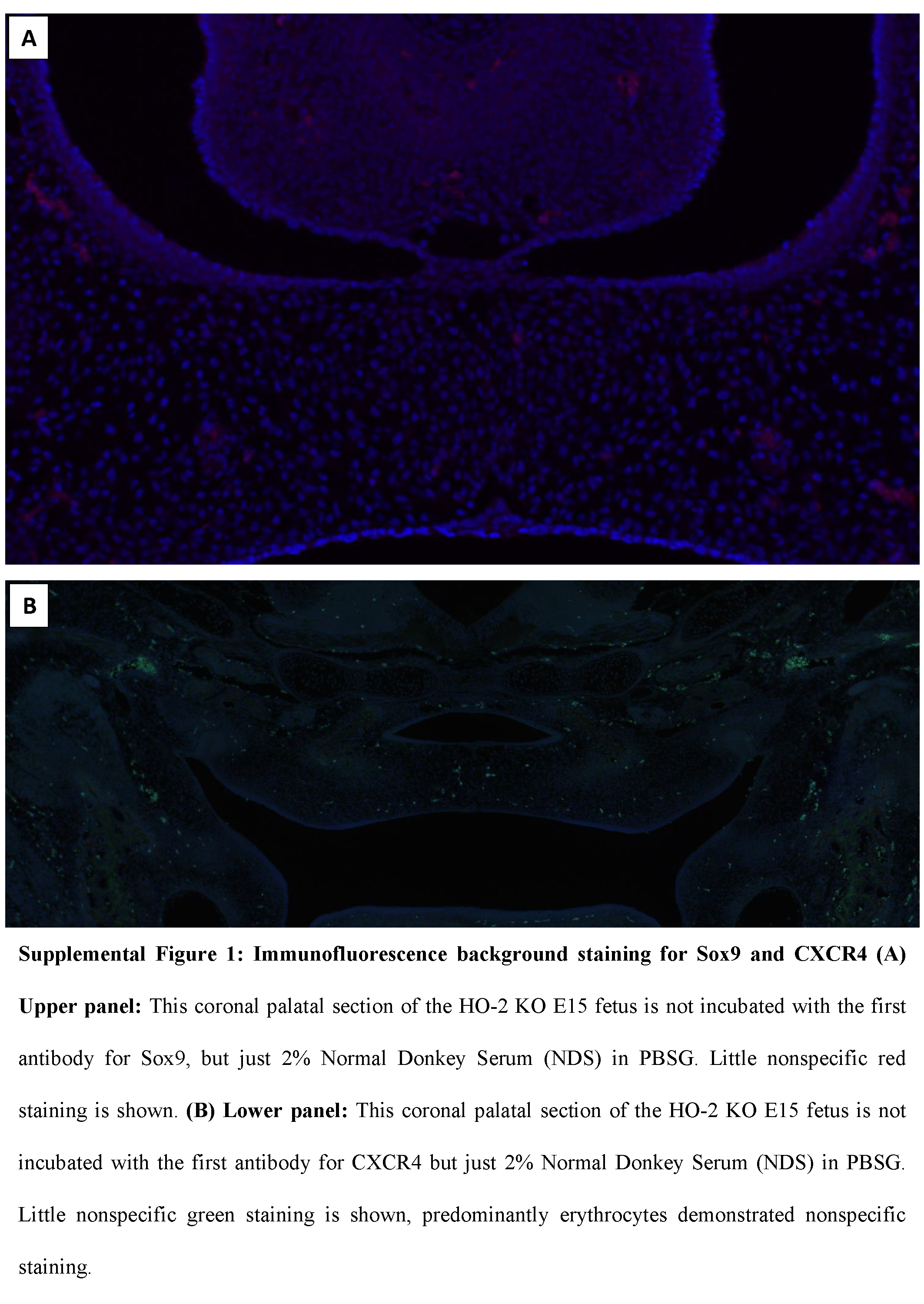

Supplement: Supplementary file 1 [file Image_1.TIFF]
